# A Novel Graphene Metal Semi-Insulator Semiconductor Transistor and Its New Super-Low Power Mechanism

**DOI:** 10.1038/s41598-019-40104-9

**Published:** 2019-03-06

**Authors:** Ping Li, R. Z. Zeng, Y. B. Liao, Q. W. Zhang, J. H. Zhou

**Affiliations:** State Key Laboratory of Electronic Thin Films and Integrated Devices, University of Electronic Science and Technology of China, Chengdu, 610054 P. R. China

## Abstract

The state-of-art Si Matel-Oxide-Semiconductor Field-Effect-Transistor (MOS-FET) meets the problem of the Power Consumption (P_C_) can not be effecively deceased guided by the Moore’s Law as before. The GFET has the problem of the device can not be effectively turned off, since the band-gap of the graphene is zero. To solve these problems, noticing the amount of the carriers in the 2 dementional semiconductor material is limited, we propose a Matel-Semi-Insulator-Semiconductor Field-Effect-Transistor (MSIS-FET) to replace the traditional MOS-FET. We verify our idea by fabricating the graphene MSIS-FETs using the natural Aluminium-oxide (Al-oxide) as the semi-insulator gate dielectric. From MSIS-FETs fabricated, we obtain following experimental results. The graphene MSIS-FET is turned off very well, a recorded high Ids on/off ratio of 5 × 10^7^ is achieved. A saddle and close-loop shape transfer feature of Ids-Vgs is obtained first time for transistors. A non-volatile memory characteristics is observed. A carrier re-injection principle and a super-Low P_C_ mechanism for semiconductor devices and integrated circuits (ICs) are found from the transfer feature of the graphene MSIS-FET. It is shown that the P_C_ of the semiconductor devices and (ICs) can be reduced by over three orders of magnitude by using this new mechanism.

## Introduction

Since the silicon (Si) MOS-FET was born, the fuction of the device gate was to induce the carriers (electrons or holes) in the semiconductor channel by applying an electric field to the channel through the gate dielcetric which was an insulator^1^. The operating principle of the Graphene-Field-Effect-Transistor (GFET) was similar to that of the Si MOS-FET, the gate dielcetric was still a insulator^2,3^. The present information technology based on the MOS-FET faces two problems which looks uncorrelated. The one is the Power Consumption (P_C_) of the Integrated Circuit (IC) is becoming a big problem, along with the information technology enters the cloud calculation and big date era, resulted from that, with the channel length or feature size of the MOS-FET scaling down, the P_C_ can only be reduced linearly changed from previous squarely^4–9^. The second is that since the band-gap of the graphene is zero, the GFET can not be effectively turned off^10–17^. We think that the research discoveries from the GFET which is made from a single atomic layer can provide the solution to the first problem, because the substrate thickness of the Si Complementary-Metal-Oxide-Semicondector (CMOS) IC is becoming thin and thin. In decades, the feature size of the CMOS ICs using Si as the semiconducor material keeps being scaled down guided by the Moore’s Law. Nowaday, the 7 nm technology has developped to the industral production^6^, the 5 nm^7^ and even 3 nm^8^ technology are in the research period. In the advanced FINFET, the channels are at the two sizes of the FIN, so the effective thickness of the Si substrate is the half width of the FIN^9^. When the FIN width goes down to 3 nm^8^, the effective thickness of the Si substrate is 1.5 nm which is less than the thickness of 3 atomic layers of Si (0.543 × 3 nm)^18^.

Many efforts were done to solve the turn-off problem of the GFET, for example, by the way of the Graphene-Nano-Ribben (GNR)^19–22^ and bilayer graphene^23–26^. But only limited sucsseses were achieved resulting GFET can not be used in digital logic Very Large Scale Integtation (VLSI) circuits up to now. Nobel prize winner, Dr. Novoselov predicted that the utility of graphene in logic circuits could be realized after 2025^11^. The highist ratio of the I_ds_ on/off was achieved by the GNR^20^ with the ratio value of 1 × 10^7^. However, the GNR is too small to be applied to the VLSI circuits which need large chip area fabricated by the semiconductor planar process. Perhaps for this reason, people turned to investigate the devices made of 2D M_O_S_2_ materials with the band-gap recently^27–31^.

Recongnizing the traditional methods to turn off the GFET had a lot of problems and noticing a fact which may be ignored by people, the number of carriers in the 2D semiconductor is much smaller than that in the 3D semiconductor, we propose a novel device structure named as the MSIS-FET correspoding to tradtional MOS-FET. Namely, the semi-insulaor is used to replace the oxided layer or the insulator in the MOS-FET. We predicted that the graphene MSIS-FET could be turned off by the postive gate voltage V_gs_ on which the electrons with negative charges in the channel would move toward the gate through the semi-insulaor gate dielectric.

Based on the analysis above, we fabricate the graphene MSIS-FET by using the natural Al-oxide as the gate dielectric. By testing the graphene MSIS-FETs fabricated, we obtain following experimental results. The graphene MSIS-FET with monolayer atom is turned off and on very well, the I_ds_ on/off ratio of 5 × 10^7^ is achieved which is about 5 times lager than the previous record of 1 × 10^7^ created by the GNR^20^. A saddle and close-loop shape transfer feature of I_ds_~V_gs_ is obtained which is obviously different from that of the semiconductor devices reported. A kind of the non-volatile memory characteristics is observed in the measurement of the graphene MSIS-FET, which gives out an important evidence to our turned-off thorey of the MSIS-FET device.

We find a carrier re-injection principle and a super-Low power mechanism from the tested features of the graphene MSIS-FET. In order to show how the super-low power mechanism works and the degree of the P_C_ reduction may be, the transfer features of the reported FINFET^8^ and the Si MSIS-FET inferred from the graphene MSIS-FET are shown.

## Theory and Experiments

### Turn-off principle of the MSIS-FET

**Figure 1:**
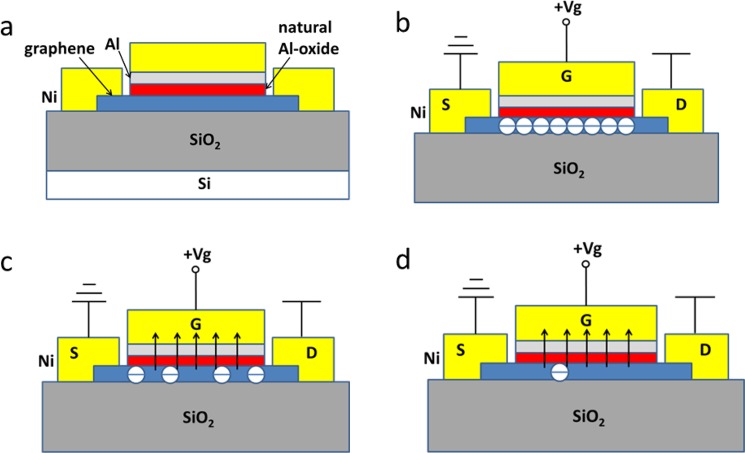
The schematic diagram of the graphene MSIS-FET and the electrons in the graphene channel with V_gs_ increasing during the turning-off procedure of the device. (**a**) The schematic diagram of the graphene MSIS-FET. (**b**) When the V_gs_ is low, a lot of electrons existing in the graphene. (**c**) When the V_gs_ is higher, less electrons existing in the grapheme. (**d**) When the V_gs_ is high enough, electrons seldom remaining in the grapheme.

The schematic diagram of the graphene MSIS-FET is shown in Fig. 1a. Instead of using a layer of insulator, such as SiO_2_ or HaO_2_, a layer of the natural Al-oxide is used as the gate dielectric. Figure 1b–d are the schematic diagrams for the gate extracting the electrons in the graphene channel with the positive gate voltage (V_gs_) increasing during the turning-off procedure of the graphene MSIS-FET.

### Fabrication

**Figure 2:**
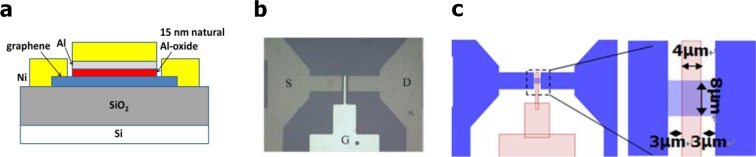
The schematic diagram of the top-gate structure graphene MSIS-FET, photograph and the layout with the key sizes. (**a**) The schematic diagram of the top-gate structure graphene MSIS-FET with 15 nm natural Al-oxide. (**b**) The typical photograph of the top-gate structure graphene MSIS-FET. (**c**) The layout and key sizes of the top-gate structure graphene MSIS-FET.

The fabrication process of the graphene MSIS-FET with the top gate is described in details in the Supplementary Information (S1). The photograph and layout with the key sizes are shown in Fig. 2.

Briefly speaking, the fabrication process of the graphene MSIS-FET with the top gate was a planar process like that for a semiconductor IC except for the transferring the graphene from a copper (Cu) substrate to the SiO_2_/Si substrate^32^.

### Tests and discussions

**Figure 3:**
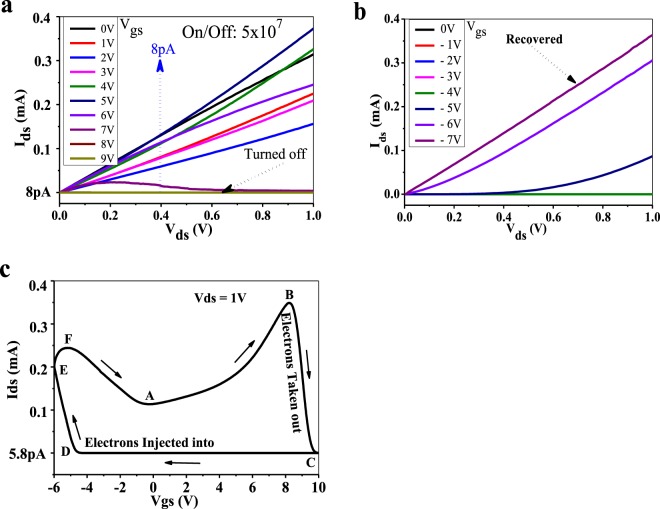
The measured features of the graphene MSIS-FET. (**a**) The turning-off output curves under the positive gate voltages. (**b**) The turning-on output curves under the negative gate voltages. (**c**) The loop shape transfer curve of Ids-Vgs.

After the fabrication, the graphene MSIS-FETs with the top gate are tested by the semiconductor parameter analysor of Agilent 4155B. Shown in Fig. 3 are the measured features of the graphene MSIS-FET. From Fig. 3a, it can be seen that when V_gs_ = 9 V, the device is turned off very well, I_ds_(off) = 8 pA, I_ds_(on) = 0.4 mA, the I_ds_ on/off ratio is 5 × 10^7^ which is about 5 times larger than the best reported ratio created by the GNR^20^. This experimental result has verifies that the graphene MSIS-FET can be turned off by a postive V_gs_.

In order to verify the turn-off characteristics of the graphene MSIS-FET was caused by using the gate dielectric of the natural Al-oxide, the compaision experiments were done by fabricating both graphene transistors with the natural Al-oxide and natural Al-oxide plus HaO_2_ respectively. Both devices have the back-gate as shown in the schematic diagrams Fig. S1A,B of the Supplementary Information (S2). The tested results are shown in Fig. S2A,B, from which it can be seen that the graphene MSIS-FET with natural Al-oxide can be turned off, while the traditional GFET with the natural Al-oxide plus HaO_2_ can not. Both devices have the same graphene material and sizes. The I_gs_~V_gs_ features of two devices are shown in Fig. S3A,B. The I_gs_~V_gs_ features of the top-gate graphene MSIS-FET is shown in Fig. S3C. From the figures, we can see that the resisitance of the 5 nm natural Al-oxide is about 1.9 × 10^3^ MΩ, the resisitance of the 15 nm natural Al-oxide is about 1.8 × 10^4^ MΩ, while the resisitance of the 5 nm natural Al-oxide plus 20 nm HaO_2_ is infinite. For the reason of the resisitance of the natural Al-oxide is quite large but it is not infinite, the natural Al-oxide is called as a semi-insulator.

After getting the results of the top gate devices in Fig. 3a and the back gate devices in Fig. S2, we observed that both top and back gate devices could not be turned on once more by any positive V_gs_ in about 24 hours. This phenomenon is similar to the performance of the floating gate non-volatile semiconductor memory, when the power supply is off, the state of device is keeping^18^. This experimental phenomenon provides a strong support to our turn-off principle because it indicates that, at this time, there are almost no electrons remaining in the graphene channel. The related tested results are shown and discussed in Supplementary Information (S3).

At this moment, observing the positive V_gs_ could not turn on the device again, we could not measure the Ids~Vgs transfer feature for the time being. We thought that if we were right, then the graphene MSIS-FET should be turned on by the negative V_gs_ injecting the electrons with the negative charges into the graphene which has a higher potential. So the negative V_gs_ was applied to the device and the tested result is shown in Fig. 3b, from which we can see that, when V_gs_ = −7 V, the conductivity of the graphene MSIS-FET is restored. The device can be immediately turned on by this way after it is turned off.

Based on the above-mentioned turning off and on of the graphene MSIS-FET, we arranged a test by changing V_gs_ from −6V to +10 V and then from +10 V to −6 V, the forward and backward I_ds_~V_gs_ current curve at V_ds_ = 1 V is shown in Fig. 3c which is called as the transfer feature of the device. For a conventional MOS-FET, the transfer feature can be tested immediately after the output feature (like Fig. 3a) is completed, however for the MSIS-FET, the transfer feature can only be obtained like the way of getting Fig. 3c. This fact, from one aspect, shows the difference between the MOS-FET and MSIS-FET.

For importance and convenience of discussions, LP point is named for the point B in Fig. 3c, (LP comes from word “Leaping” which means I_ds_ begins to jump down). The V_gs_ corresponding to the LP point is named V_LP_. The appearance of the LP point for our graphene MSIS-FET is the result of the balance between the functions of the gate extracting electrons and inducing electrons. When V_gs_ is higher than the V_LP_, the function of the gate extracting electrons is stronger than that of inducing electrons, as a result, the I_ds_ gets deceasing, until V_gs_ reaches the value corresponding to the point C, the graphene MSIS-FET is turned off.

### Super-low power mechanism

When we obtained the close-loop shape transfer feature of the graphene MSIS-FET shown in Fig. 3c, we understood that the turn-on procedure of the graphene MSIS-FET was a procedure of the carrier re-injection. This new carrier re-injection principle could be used to form the super-low power semiconductor devices and ICs, because, on the line DE in the Fig. 3c, people can chose any point to end the carrier re-injection. The point chosen by people is the closer to the point D, the smaller the I_ds_ on-state will be.

**Figure 4:**
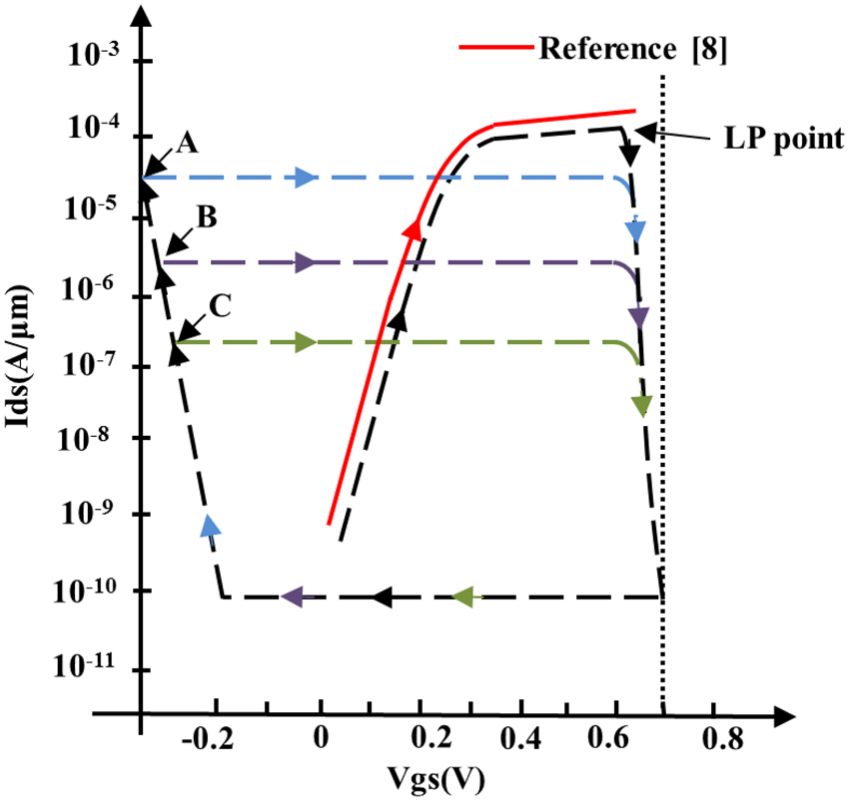
The diagram for description the super-low power mechanism of the MSIS-FET.

In order to explain more clearly how the super-low power mechanism works, Fig. 4 is drawn. In the figure, the transfer features of the 3 nm FINFET comes from the ref. ^8^. The performance of the Si MSIS-FET shown in Fig. 4 is inferred from the tested feature of our graphene MSIS-FET at the assumptions of the Si MSIS-FET has the same sizes as the 3 nm FINFET. Another assumption is that the Si MSIS-FET can be turned off in the same way as the graphene MSIS-FET.

Shown in Fig. 4 by the red line is the performance of the 3 nm N type Si FINFET^16^, it can be seen that if the V_ds_ is defined as 0.7 V, the I_ds_ is fixed at 2 × 10^−4^ A. The black line in Fig. 4 shows the turn-off and turn-on procedure of the Si MSIS-FET. At the beginning of the V_gs_ rising, the I_ds_ rises like the AB line in Fig. 3c. When the V_gs_ reaches the V_LP_, the I_ds_ reaches its maximum. When the V_gs_ exceeds V_LP_, the I_ds_ deceases rapidly, until V_gs_ reaches 0.7 V, the I_ds_ reaches its minimum, the device is turned off. The blue, purple and green lines indicate that by choosing different negative gate voltages during the turning on procedure, people can get the I_ds_ of the Si MSIS-FET, I_dsMSIS-FET_, equaling to 1/10 (the blue line), 1/100 (the purple line) or 1/1000 (the green line) of 2 × 10^−4^ A. As a result, the P_C_ can be reduced by orders of magnitude, since P_C_ = V_ds_ × I_dsMSIS-FET_. Therefore, the reduction of P_C_ of the Si MSIS-FET is in a leaping or revolutionary way.

The importance of the amount of P_C_ reduction discussed above can be understood by reviewing the way of the P_C_ reduction for a traditional Si MOS-FET guided by the Moore’s Law. When the feature size is scaled down by a fact of 2, the P_C_ can only be reduced by 2~4 times^4^.

From Fig. 4, it can be seen that, for the Si MSIS-FET, the transfer feature has a shape close to the parallelogram. While for the graphene MSIS-FET, the transfer feature has a saddle shape as shown in the Fig. 3c. We predict that the transfer features of the MSIS-FETs made from 2D materials with band-gap such as MoS_2_ and black phosphorus will have the same shape as in Fig. 4. The difference between the features of the MSIS-FETs with and without the band-gap can be predicted as following.

Since the existance of the Dirac point in the graphene MSIS-FET, before the Vgs equals to the Dirac Voltage V_Dirac_, the operating carriers are holes, after the Vgs equals to the V_Dirac_, the operating carriers are electrons^3^, however, for the MSIS-FETs with the band-gap, for example, for the Si NMOS, the operating carriers are electrons at all the time. In short, since no Ids mimium point caused by the Dirac point existing in the Si MSIS-FET, the saddle shape will not appear in its transfer feature. It needs to point out that the top edge of the parallelogram may not be horizontal. It may has a positive or negative slope depended on which fuction of the gate extracting and inducing the carriers is stronger. If the extracting one is stronger, the top edge will be down to the left before the LP point. Reversely, if the inducing one is stronger, then the top edge will be up to the left before the LP point. The other 2D material MSIS-FETs beside the graphene MSIS-FET may also be important applications of the MSIS-FET, because they can also realize the super-low power based on the machanism found in this paper.

## Conclusion

In this paper, we propose and experimentally demonstrate the graphene MSIS-FET. The graphene MSIS-FET is turned off and on very effectively by changing the polar of the V_gs_. The saddle and close-loop transfer feature of the graphene MSIS-FET is measured first time. The non-volatile memory feature of the graphene MSIS-FET is observed which provides an evidence to our turn-off principle and may be used to form a new type semiconductor memory products.

The contribution of this paper to graphene electronics is that the planar fabrication process of the graphene MSIS-FET may let the graphene material be used much easier in the future. The most important contribution of this paper is the discoveries of the carrier re-injection principle and the super-low power mechanism of the graphene MSIS-FET by which the amount of electrons or holes in the semiconductor channel can be controlled first time by the people. The scientific significace of the MSIS-FET verified by our graphene MSIS-FET is that it makes people changing from passtively accepting the carrier amount in the MOS-FET channel fixed by the operating voltage to actively adjusting the carrier amount in the MSIS-FET channel, as a result, it creates a new way to realize the extremely low power semiconductor devices and ICs.

## Supplementary information

Supplementary Information for A Novel Graphene Metal Semi-Insulator Semiconductor Transistor and Its New Super-Low Power Mechanism
