# Supplementary material for: A Novel Graphene Metal Semi-Insulator Semiconductor Transistor and Its New Super-Low Power Mechanism
Source: Sci Rep. 2019 Mar 6;9:3642. doi: 10.1038/s41598-019-40104-9 (PMC6403360; doi:10.1038/s41598-019-40104-9)
Supplement: Supplementary file 1 — Supplementary Information for A Novel Graphene Metal Semi-Insulator Semiconductor Transistor and Its New Super-Low Power Mechanism [file 41598_2019_40104_MOESM1_ESM.doc]

Supplementary Information

A Novel Graphene Metal Semi-Insulator Semiconductor Transistor and Its New Super-Low Power Mechanism

Ping Li*， R.Z. Zeng，Y.B. Liao*，Q.W. Zhang，J.H. Zhou

State Key Laboratory of Electronic Thin Films and Integrated Devices，

University of Electronic Science and Technology of China

*e-mail:pli@uestc.edu.cn; lyb@uestc.edu.cn

**S1.The fabrication process of the graphene Metal-Semi-Insulator-Semiconductor Field-Effect-Transistor (MSIS-FET) device with the top-gate**

The fabrication process of the graphene MSIS-FET with the top-gate is described in details as following in sequence. The start substrate is a high-resistivity Si wafer with the resistivity of 10000 Ω•cm. A layer of 1µm thickness SiO_2_ is grown on the substrate by the high temperature oxidation. The single atomic layer of the graphene grown by the CVD is transferred to the surface of the SiO_2_/Si. The growth and transfer process is similar to that in the paper^32^. The graphene is defined by the plasma etching of the oxygen. The standard photolithography process is used to define gate dielectric and gate electrode metal. A layer of 5 nm Aluminum (Al) is deposited on the surface of the sample by the electron beam evaporator. The Al is oxidized to the natural Aluminum-oxide (Al-oxide) when the die of the sample is exposed in the air of the class 1000 cleaning-room for 10 hours with the temperature of 21℃. After this, another two layers of the 5 nm Al are deposited and oxidized respectively. The gate dielectric is completed in this way. Then, the gate metal electrode is formed by the deposition and lift-off of the 20 nm Al and 20 nm Ni. After another photolithography process, the drain and source metal electrodes are formed by the deposition and lift-off of the 40 nm nickel (Ni). The length and width of the channel are 4 µm and 8µm. The distance between the source and drain electrodes is 10µm. All of the transistors are tested by Agilent 4155B Semiconductor Parameter Analyzer in the atmosphere at the room temperature. The photograph and layout with the key sizes are shown in Fig.2 of this paper.

**S2.The fabrication process of the graphene MSIS-FET and GFET with the back-gate and tested results**

The fabrication process of the graphene MSIS-FET is as following. As same as for the top device, the start substrate is SiO_2_/Si. A trench of 300nm depth is etched on the surface of the SiO_2_ by a reactive ion etching (RIE) with the gases of the CHF_3_, Ar and He. A layer of 400nm Al is deposited on the surface of the substrate by the electron-beam evaporation. The Al out of the trench is removed, and the surface is smoothed by the Chemical-Mechanical-Polishing (CMP). A layer of the 5nm natural Al-oxide grows on the surface of the Al electrode while it is exposed in the air^13^. The single atomic layer of the graphene grown by the CVD is transferred to the surface with the gate electrode and gate dielectric. The graphene is defined by the oxygen plasma etching. A layer of 40nm metal Ni is deposited by the electron-beam evaporation and lifted off to shape the source and drain electrodes. In this way, the fabrication of the graphene MSIS-FET transistor with the buried-back-gate is completed. The channel length and width are 3µm and 20µm respectively, the distance between the source and drain metals is 8µm. The device structure of the buried-back-gate is shown in Fig.S1 A. The photo and the channel dimensions are shown in Fig.S1 C, and Fig.S1 D. In order to verify the turn-off characteristics of the graphene MSIS-FET was caused by using the gate dielectric of the natural Al-oxide, the comparison experiment was done by fabricating the GFET with the same sizes and materials except that the GFET has a layer of 20 nm HfO_2_ deposited on the natural Al-oxide. The schematic diagram of the later is shown in Fig.S1 B.

The tested output features of the two devices are shown in Fig.S2 A and Fig.S2 B. From Fig.S2 A, it is clear, when the gate voltage V_gs_ is 5V, a turn-off characteristics is obtained with the I_ds_ on/off ratio of 10^5^. In contrast, from Fig.S2 B, it can be seen, the GFET with natural Al-oxide + HfO_2_ gate dielectric can not be turned off, the I_ds_ on/off ratio of it is just 1.4. The measured I_gs_~V_gs_ curves for the two devices are shown in Fig.S3 A and B. It can be seen, when the V_gs_ is larger (V_gs_≥3V), the resistance of the natural Al-oxide is obviously reduced, but that of the natural Al-oxide + HfO_2_ keeps almost infinity. The measured I_gs_~V_gs_ curve for the top-gate device is shown in Fig.S3 C, which is similar to Fig. S3 A. The results of Fig.S2 and Fig.S3 verify that the turn-off of the graphene MSIS-FET relies on the conductance of the natural Al-oxide dielectric. It is proved that if the conductance of the natural Al-oxide dielectric is blocked by the adding layer of HfO_2_, then the device can not be turned off any more.


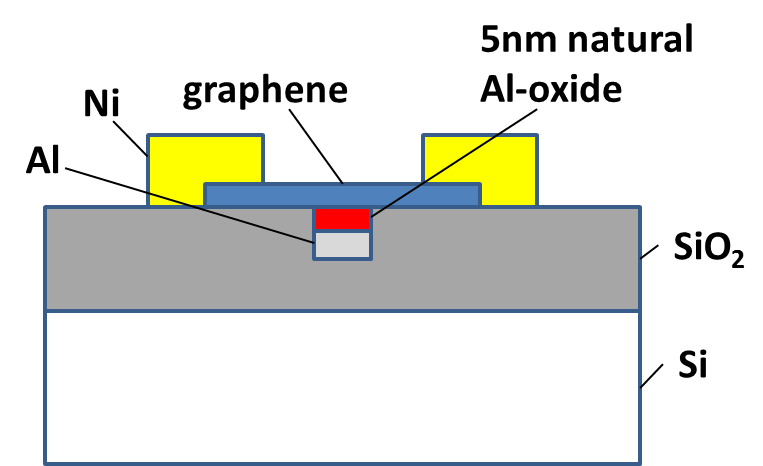


A


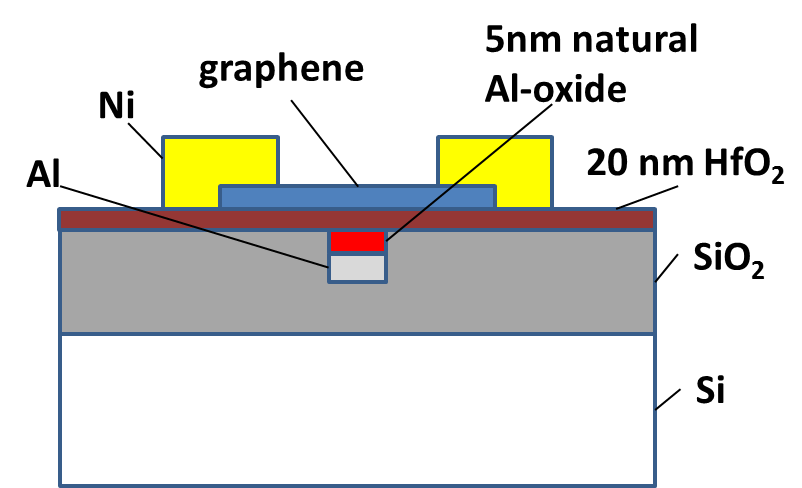


B

C


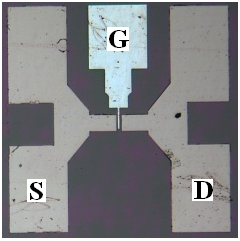

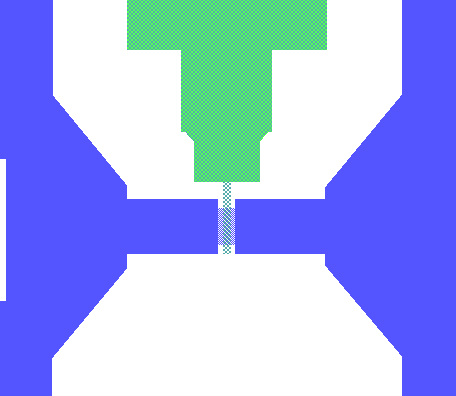

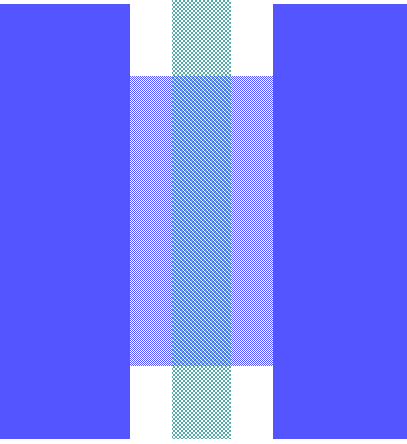


**20μm**

**4μm**

**3μm**

**3μm**

D

Fig.S1 The schematic diagram of the back-gate structure graphene MSIS-FET and GFET, photograph and the layout with the key sizes.

A. The schematic diagram of the back-gate structure graphene MSIS-FET with 5nm natural Al-oxide dielectric.

B. The schematic diagram of the back-gate structure GFET with 5nm natural Al-oxide + 20nm HfO_2_ gate dielectric.

C. The typical device photograph of the back-gate structure devices.

D. The layout and key sizes of the back-gate structure devices.

Fig.S2 The output characteristics of the graphene MSIS-FET and GFET with the back-gate structure.

A. The output characteristics curves of the graphene MSIS-FET with 5nm natural Al-oxide gate dielectric.

B. The output characteristics curves of the GFET with 5nm natural Al-oxide + 20nm HfO_2_ gate dielectric.

Fig. S3 The I_gs_~V_gs_ curves of the graphene MSIS-FETs and GFET.

A. The curve of I_gs_~V_gs_ for the back-gate graphene MSIS-FET with 5nm natural Al-oxide gate dielectric from which the gate resistance of about 1.9×10^3^ MΩ is calculated.

B. The curve of I_gs_~V_gs_ for the back-gate GFET with the 5nm natural Al-oxide + 20nm HfO_2_ gate dielectric from which the gate resistance of infinity is seen.

C. The curve of I_gs_~V_gs_ for the top-gate graphene MSIS-FET with 15nm natural Al-oxide gate dielectric from which the gate resistance of about 1.8×10^4^ MΩ is calculated.

**S3.The non-volatile memory performance of the graphene MSIS-FET**

After getting the results of the top gate devices in Fig.3a and the back gate devices in Fig.S2, we observed that both top and back gate devices could not be turned on once more by any positive V_gs_ in about 24 hours. This phenomenon is reasonable as can be seen from Fig.3c. It is clear that, after the V_gs_ reached the C point, no matter, V_gs_ changes from 0V to 9V or from 9V to 0V, the graphene MSIS-FET can not be turned again. We tried to applied the 0V to 9V V_gs_ once again immediately after the device is turned off , what we got is shown in Fig. S4, it verified that the device could not be turned on by 0~9V V_gs_ in this way. It is very interesting that, we observed the device can be turned on once gain by 0~9V V_gs_, after it being kept in our clean-room for 24 hours. The out-put curves of I_ds_~V_ds_ are shown in Fig. S5 from which the conductance of the device recovers naturally after the 24 hours. This result suggests that the graphene MSIS-FET may has a potential to be used as a new type of non-volatile semiconductor memory. This phenomenon is similar to the performance of the floating gate non-volatile semiconductor memory, when the power supply is off, the state of device is keeping^1^. The advantage of the non-volatile memory of the MSIS-FET is that the MSIS-FET needs not the floating gate. It is well known that the floating gate products are much expensive than the CMOS products for the reason of additional a few masks are needed by the former. And the testing procedrue of the former is much complex.

Fig.S4 The transfer characteristic curve of the graphene MSIS-FET measured immediately after the device is turned off first time.

Fig.S5 The out-put characteristic curve of the graphene MSIS-FET tested second time, 24 hours after the device is turned off first time.
